# Supplementary material for: Multi-Modal Imaging to Assess the Follicular Delivery of Zinc Pyrithione
Source: Pharmaceutics. 2022 May 17;14(5):1076. doi: 10.3390/pharmaceutics14051076 (PMC9145647; doi:10.3390/pharmaceutics14051076)
Supplement: Supplementary file 1 [file pharmaceutics-14-01076-s001.zip › pharmaceutics-1733814-supplementary.pdf]

pharmaceutics

Multi-Modal Imaging to Assess the Follicular Delivery of Zinc Pyrithione

Sean E. Mangion [1,2,3], Lydia Sandiford [1,2,†], Yousuf Mohammed [4], Michael S. Roberts [1,2,4] and Amy M. Holmes [1,2,*]

Table S1. Ingredients in the three commercial anti-dandruff shampoos.

Ingredient Class	Head & Shoulders (H&S)	T/Gel	Cedel
**Active**	ZnPT 1% w/v	ZnPT 1% w/v	ZnPT 2% w/v
**Solvent**	Water	Water	Water
**Surfactants / foam boosters**	Sodium Lauryl Sulfate, Sodium Laureth Sulfate, Cocamidopropyl Betaine	Sodium Laureth Sulfate, Cocamidopropyl Betaine, Ammonium Lauryl Sulfate, Sodium Methyl Cocoyl Taurate	Sodium Laureth Sulfate, Cocamide DEA, Sodium Lauryl Sulfate, Cocamide MEA, Sodium Methyl Cocoyl Taurate
**Dispersing agents**	Glycol Distearate	C12-13 Pareth-3, C12-13 Pareth-23	Glycol Distearate, Bentonite
**Conditioning agents**	Dimethicone	Divinyldimethicone/Dime-thicone, Castor Isostearate, Succinate, PG Propyl Silanetriol, Amodimethicone, Cetearyl Alcohol, Ceteareth-20	
**Deposition enhancer**	Guar Hydroxypropyltrimoniu-m Chloride	Guar HydroxypropyltrimoniumChloride	
**pH adjustment**	Hydrochloric Acid	Sodium Hydroxide	Lactic Acid
**Rheology modifiers**	Sodium Chloride, Sodium Xylenesulfonate	Sodium Chloride, Acrylates Copolymer	Sodium Chloride
**Preservatives**	Sodium Benzoate, Methylchloroisothiazoli-none, Methylisothiazolinone	DMDM Hydantoin	Methylchloroisothiazolinone, Methylisothiazolinone
**Colour/ fragrance**	Parfum, Hexyl Cinnamal, Linalool, CI 42090, CI 17200	Blue, 1 Fragrance	Fragrance, Cl 42090, Cl 19140
**Miscellaneous**	Zinc Carbonate, Magnesium Carbonate, Hydroxide	Hydrolyzed Wheat Protein, Tocopheryl Acetate	

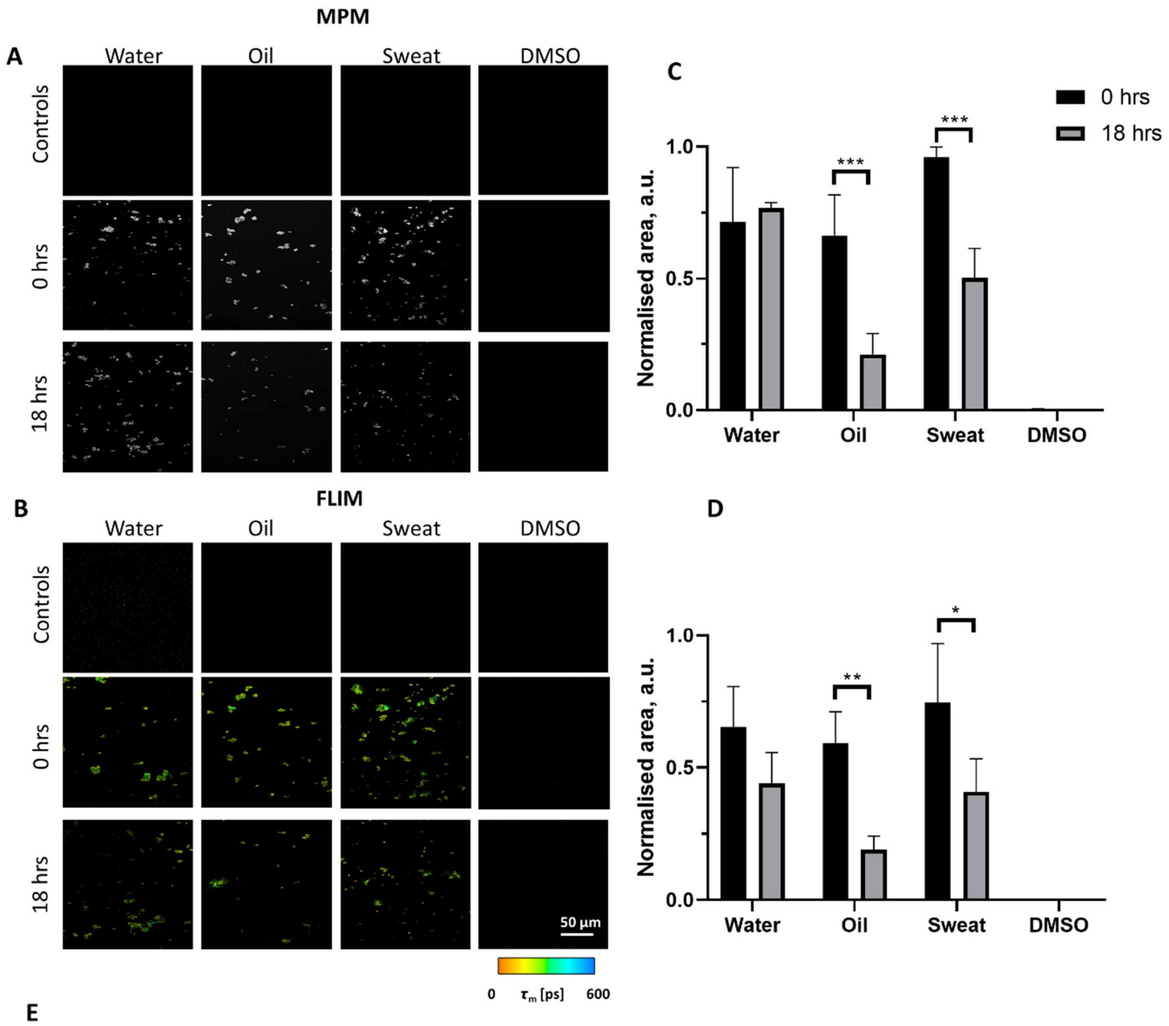

E

Solvent	τm (ps)	
	0 hrs	18 hrs
**Water**	180 ± 23	218 ± 15
**Olive oil**	203 ± 17	196 ± 15
**Artificial sweat**	181 ± 44	213 ± 9

Figure S1. Dissolution of ZnPT in oil and artificial sweat**.** (**A**) Time course MPM imaging and (**B**) FLIM imaging, demonstrating dissolution over 18 h when incubation at 34°C. (**C**) corresponding intensity measurements for MPM images and (**D**) FLIM images respectively and (**E**) lifetime analysis. * Corresponds to $p<0.05$, ** $p<0.01$ and *** $p<0.001$.

Multi-modal imaging of ZnPT on skin

Initial FLIM imaging after aqueous ZnPT application is shown in supplementary Figure 2. Sequential z-stacks were obtained from the surface over the hair follicle to 90 µm. In the treated sample, short lifetime particles are visible adhering to the hair fiber surface and at the follicle entrance (Supp Fig 2b, white arrows). They can also be observed up to 90 µm. A trace of a particle cluster is shown in Supplementary Fig 2c, corresponding to ZnPT. While ZnPT particles are visible, poor skin contrast due to limited passive permeation of the acriflavine dye makes it difficult to establish spatial delivery location.

Skin sections proved to be more reliable to image in terms of both the morphology of skin and spatial delivery of ZnPT particles. Zinpyr-1 is a zinc labile dye that provided excellent skin contrast. Fig 5d shows skin cryosections stained with Zinpyr-1. Particles of ZnPT are visible at the entrance to a skin furrow in the 395-420 emission channel. MPM

however is not as sensitive as FLIM, demonstrated by the control section, which detected endogenous skin fluorophores (melanin granules at the dermal epidermal junction, collagen within the dermis fibers exhibiting second harmonic generation) in the same spectral channel. Skin sectioning with Zinpyr-1 was used in the remaining experiments to assess delivery.

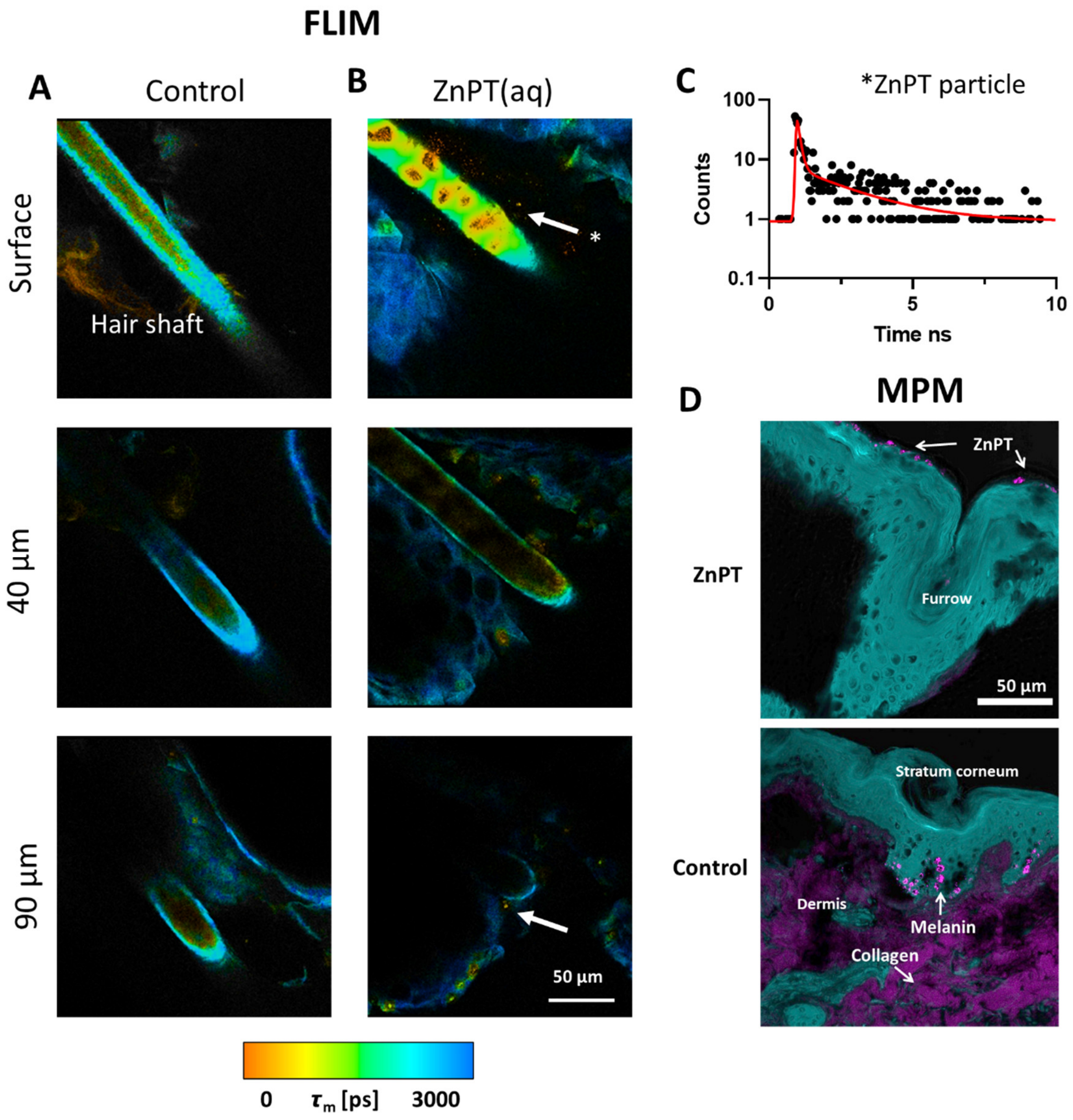

Figure S2. Multi-modal imaging of ZnPT on skin. (**A**) FLIM z-stacks through control human hair follicle stained with acriflavine and (**B**) dosed with 2% w/v ZnPT suspension. Two-photon excitation at 740 nm with emission collected using a 405/10 nm band-pass filter. Arrows indicate location of ZnPT particles. (**C**) Corresponding decay curve of particle on surface. (**D**) MPM of Zinpyr-1-stained skin cryosections showing detection of ZnPT on surface. Two-photon excitation at 800 nm with emission collected at 395 – 420 nm (pink); and one-photon excitation at 488 nm with emission collected at 520 – 560 nm (blue).

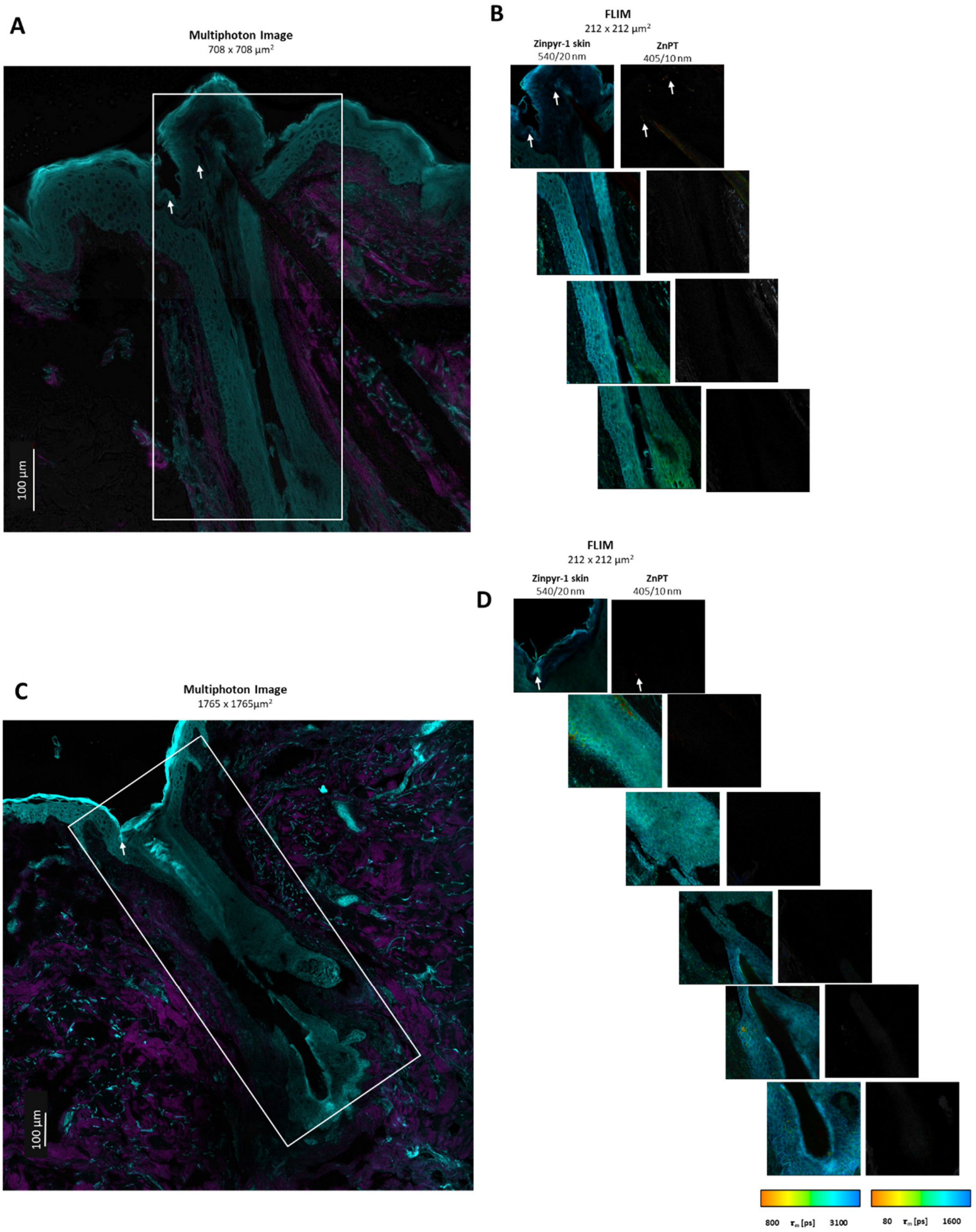

Figure S3. Follicular delivery of ZnPT from H&S. (**A**) MPM image of H&S (ZnPT 1% w/v) applied with no massage and (**B**) the corresponding FLIM images along the length of the follicle. (**C**) MPM image of H&S applied with 2-minute massage and (**D**) the corresponding FLIM images along the length of the follicle. For MPM images (**A, C**) two excitation wavelengths were used: two-photon excitation at 800 nm with emission collected at 395 – 420 nm (pink); and one-photon excitation at 488 nm with emission collected at 520 – 560 nm (blue). The parameters for the FLIM images (**B, D**) were: two-photon excitation at 740 nm with emission collected using a 405/10 nm bandpass filter for ZnPT and 540/20 nm bandpass filter for Zinpyr-1. FLIM images are pseudo coloured to the average time-weighted lifetime τ_m [ps].

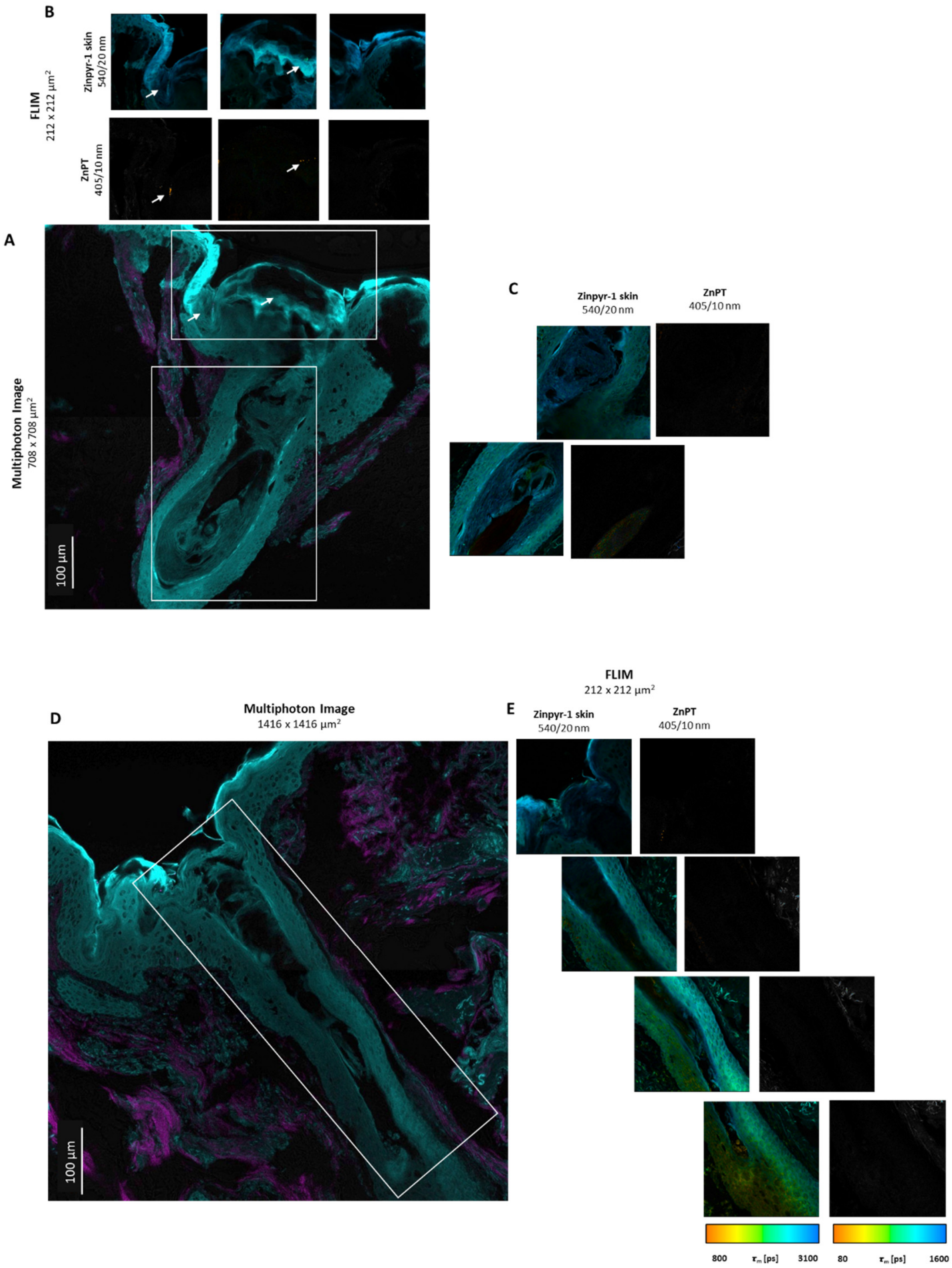

Figure S4. Follicular delivery of ZnPT from Cedel**.** (**A**) MPM image of Cedel (ZnPT 2% w/v) applied with no massage and (**B**) and (**C**) the corresponding FLIM images along the length of the follicle. (**D**) MPM image of H&S applied with 2-minute massage and (**E**) the corresponding FLIM images along the length of the follicle. For MPM images (**A, D**) two excitation wavelengths were used: two-photon excitation at 800 nm with emission collected at 395 – 420 nm (pink); and one-photon excitation at 488 nm with emission collected at 520 – 560 nm (blue). The parameters for the FLIM images (**B, C, E**) were: two-photon excitation at 740 nm with emission collected using a 405/10 nm bandpass filter for ZnPT and 540/20 nm bandpass filter for Zinpyr-1. FLIM images are pseudo coloured to the average time-weighted lifetime τ_m [ps].

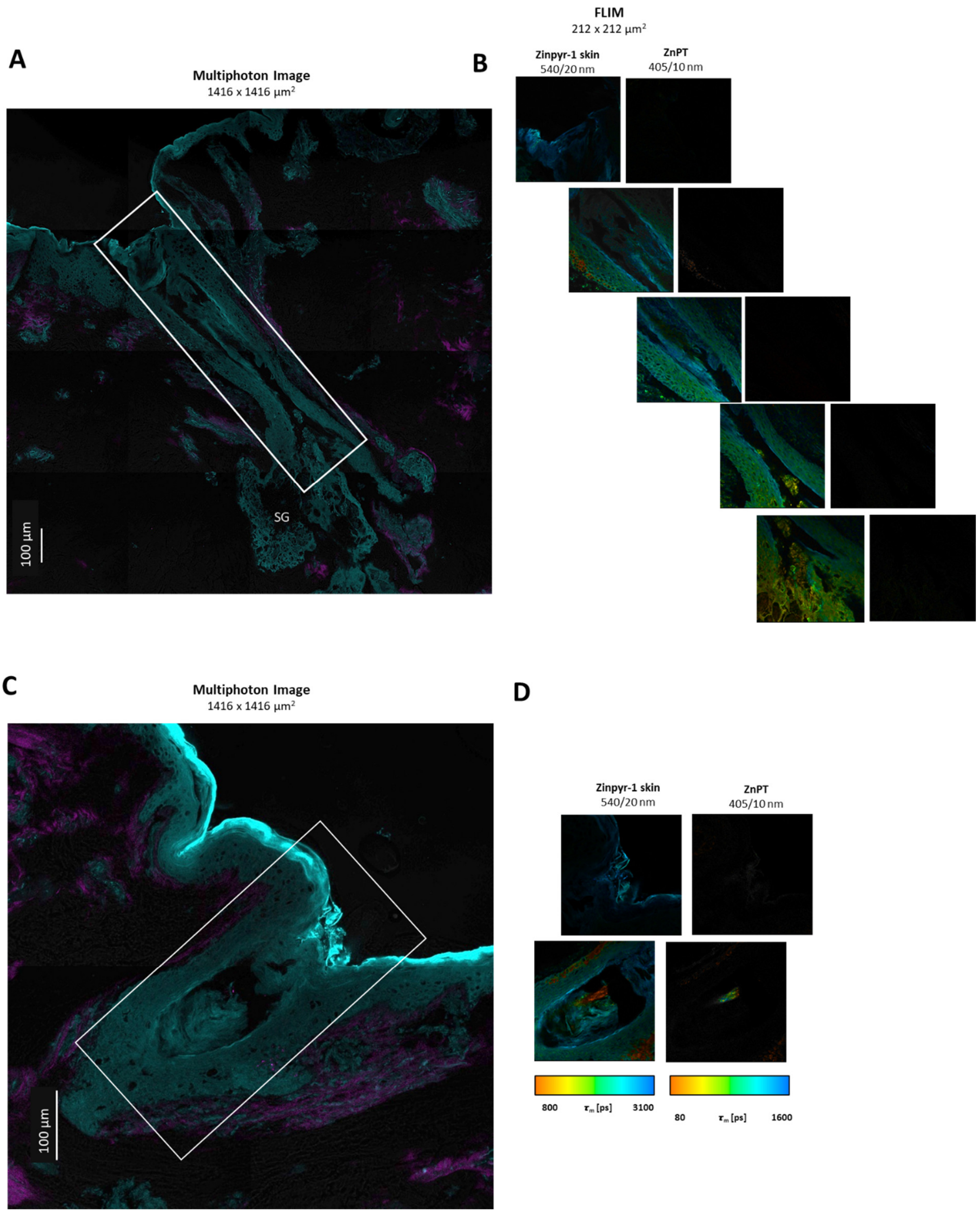

Figure S5. Follicular delivery of ZnPT from T/Gel. (**A**) MPM image of T/Gel (ZnPT 1% w/v) applied with no massage and (**B**) the corresponding FLIM images along the length of the follicle. (**C**) MPM image of H&S applied with 2-minute massage and (**D**) the corresponding FLIM images along the length of the follicle. For MPM images (**A, C**) two excitation wavelengths were used: two-photon excitation at 800 nm with emission collected at 395 – 420 nm (pink); and one-photon excitation at 488 nm with emission collected at 520 – 560 nm (blue). The parameters for the FLIM images (**B, D**) were: two-photon excitation at 740 nm with emission collected using a 405/10 nm bandpass filter for ZnPT and 540/20 nm bandpass filter for Zinpyr-1. FLIM images are pseudo coloured to the average time-weighted lifetime τ_m [ps]. SG = sebaceous gland
